# Diffusion-weighted imaging hyperintensities during the chronic stage of intracerebral hemorrhage with surgery: A new clinical situation or post-surgery artifact?

**DOI:** 10.3389/fneur.2022.948828

**Published:** 2022-09-16

**Authors:** Xiaoyan Chen, Ying Li, Shengli Guo, Xun Han, Ruozhuo Liu, Chenglin Tian, Rongtai Cui, Zhao Dong, Shengyuan Yu

**Affiliations:** ^1^Department of Neurology, First Medical Centre of Chinese PLA General Hospital, Beijing, China; ^2^Department of Radiology, First Medical Centre of Chinese PLA General Hospital, Beijing, China; ^3^Department of Neurosurgery, First Medical Centre of Chinese PLA General Hospital, Beijing, China

**Keywords:** diffusion-weighted imaging, chronic stage, intracerebral hemorrhage, surgery, extracellular methemoglobin

## Abstract

**Background and objective:**

Diffusion-weighted imaging (DWI) hyperintensities were occasionally seen at previous hematoma in patients several months after intracerebral hemorrhage with surgery. Whether they are newly occurred clinical situations or post-surgery changes is unknown. This study aims to investigate the prevalence and possible mechanisms for this phenomenon.

**Methods:**

We retrospectively reviewed the MRI database for intracerebral hemorrhage with surgery after 3 months of disease onset in our hospital. We also prospectively performed repeated multimodal MRI scans for two patients at the chronic stage after surgery for intracerebral hemorrhage.

**Results:**

We found that 14 out of 23 patients (60.9%) had DWI hyperintensities at the site of previous hematoma 3 months after intracerebral hemorrhage with surgery. All the DWI lesions were hyperintense on T1- and T2-weighted imaging, most of which appeared long and narrow in shape. The DWI lesions were usually located adjacent to the thin wall of the previous hematoma cavity close to the lateral ventricle. They were more associated with the basal ganglia hemorrhage than with the lobar hemorrhage (*P* = 0.02) and were more frequently seen for those with intraventricular hemorrhage than without (*P* = 0.02). Prospectively repeated MRI exams of two patients revealed unchanged DWI hyperintensity during the 18- and 2-month follow-up, respectively.

**Conclusion:**

The DWI lesions at previous hematoma were commonly seen in patients after surgery for intracerebral hemorrhage at the chronic stage which would persist for years. We hypothesized a possible mechanism by which extracellular methemoglobin “islands” are formed with delayed or no absorption by macrophages from adjacent thin residual brain tissue. Unnecessary further examinations and treatment would be avoided by realizing this imaging phenomenon.

## Introduction

For patients with intracerebral hemorrhage (ICH) (1), DWI hyperintensity is present at the hyperacute and late subacute (8 days to 1 month) stages (2). At the chronic stage of ICH (over 1 month), the DWI signal intensity of the hematoma usually appears as hypointense or as an isointense center with a hypointense rim (2). Distant small DWI lesions occur in approximately 10~45.9% of patients with acute ICH (1, 3–5) and in a similar rate of patients with nonacute ICH (1), which is generally considered acute microinfarct or even microbleeds of the late subacute stage and correlated with microangiopathy (6).

Patients with a large amount of ICH often undergo surgery to evacuate the hematoma. Some of these patients are quite disabled even after surgery and some may have post-stroke epilepsy (7). Patients of ICH with surgery (ICH–WS) at the chronic stage might take brain MRI examination, especially when the first attack of post-stroke epilepsy occurred. In our clinical practice, DWI hyperintense lesions were occasionally observed at the location of previous intracerebral hematoma several months after invasive surgery, which may yield confusion to doctors regarding whether they are new ischemic or hemorrhagic strokes. To the best of our knowledge, there are no studies on DWI lesions at the chronic stage of ICH–WS. In this study, we aimed to investigate the prevalence and characteristics of DWI lesions at the site of the previous hematoma for the chronic stage of ICH–WS and speculate on possible mechanisms by searching the MRI database in our hospital and prospectively performing repeated brain MRI scans for two patients with ICH–WS.

## Materials and methods

### Study population

We conducted a retrospective review of patients with ICH–WS who underwent brain MRI scans at PLA General Hospital from January 2015 to February 2022. Patients were included in this study if they had spontaneous ICH and invasive surgery for the hematoma, and MRI DWI scans were performed at least 3 months after ICH onset. Patients were excluded if they did not have MRI DWI scans at least 3 months after ICH onset and if they had isolated intraventricular hemorrhage, subarachnoid hemorrhage, subdural hematoma, or secondary causes of ICH such as vascular malformation, trauma, venous infarction, hemorrhagic transformation of ischemic infarction, or tumor. We also prospectively conducted repeated MRI scans for two patients.

### Standard protocol approvals, registrations, and patient consent

This study was approved by the local ethics committee of the Chinese PLA General Hospital. The written informed consent to participate in this study was signed by the patients or their authorized representatives.

### Data collection

The ICH was confirmed by CT or MRI at baseline. We retrieved baseline demographic, clinical, and imaging information, including age, sex, comorbid conditions, hematoma location, and volume, whether accompanied by intraventricular hemorrhage, the interval from ICH onset to surgery and MRI scan, surgery hospital, and bleeding amount during surgery.

### MRI acquisition and analysis

All the included patients underwent MRI scans at least 3 months after ICH onset. MRI examinations were performed using a 3.0 Tesla scanner (Discovery MR 750, GE Healthcare, Waukesha, Wisconsin, USA) equipped with a standard 8-channel phase array head coil or a 1.5 Tesla scanner (Signa HDxt, GE Healthcare, Waukesha, Wisconsin, USA). The 3.0 Tesla MRI protocol consisted of axial fast spin echo (FSE) T2-weighted imaging (T2WI) [repetition time (8)/echo time [TE] = 4,526/111 ms, slice thickness/gap = 5.0/1.0 mm, field of view [FOV]= 240 × 240 mm, matrix = 288 × 224], coronal fluid-attenuated inversion recovery (FLAIR) T2WI (TR/TE/inversion time[TI] = 8,500/162/2,100 ms, slice thickness/gap = 5.0/1.0 mm, FOV =240 × 240 mm, matrix = 288 × 224), and axial FLAIR T1-weighted imaging (T1WI) (TR/TE/TI = 1,850/24/780 ms, slice thickness/gap = 5.0/1.0 mm, FOV = 240 × 240 mm, matrix = 352 × 256). Diffusion-weighted imaging (DWI) (TR/TE = 3,000/65.6 ms, slice thickness = 4 mm, FOV = 240 × 240 mm, matrix = 128 × 128). Paramagnetic gadolinium-based contrast medium (0.1 mmol/kg) was injected at a rate of 4.0 ml/s, followed by the administration of a 20 ml saline bolus for some patients. Anatomical images of the whole brain were acquired.

The DWI lesions [also named as DWI-positive or DWI (+) in this article] were defined as high-signal intensity lesions on DWI accompanied by low-signal intensity on ADC. The presence of lesions on DWI was determined by one experienced neurologist (XC) and 1 neuroradiologist (YL). The characteristics of DWI lesions were described. For the patients who had MRI scans more than once, we put the imaging data of the first scan after 3 months of disease onset into analysis and recorded the longitudinal changes for DWI lesions.

For one patient who was prospectively followed up, MRI scans were performed every 6 months during the following 18 months for a total of 4 times. Apart from routine MRI scans using a 3.0 Tesla scanner as mentioned earlier, susceptibility-weighted imaging (SWI), perfusion-weighted imaging (PWI), and magnetic resonance spectroscopy (MRS) were also conducted to obtain more detailed information. The characteristics of the MRI manifestation were described for this patient. Another prospective followed-up patient took a repeated MRI scan 2 months after the first scan.

### Statistical analysis

Baseline clinical and imaging variables were compared for patients with DWI (+) vs. DWI (–). Mean values and SDs for numerical data were calculated for normally distributed data, and the median value (interquartile range, IQR) was used if the data were not normally distributed. Frequencies with absolute numbers and percentages were presented for categorical data. Differences in numerical data (age, hematoma volume, duration from ICH onset to surgery and MRI scan, bleeding amount during surgery) were assessed using independent *t*-tests for parametric or the Mann–Whitney tests for nonparametric data. Pearson chi-square or Fisher's exact tests were used to compare the frequencies of categorical data (sex, comorbid conditions, whether accompanied by intraventricular hemorrhage, and surgery hospital). Odds ratios (ORs) with a 95% (CI) were calculated. A *P-*value ≦0.05 was considered to be statistically significant using two-sided tests. All the statistical analyses were carried out using IBM SPSS 26.0 (Chicago, IL, USA).

## Results

### Characteristics of ICH–WS with and without DWI hyperintensities

**Figure 1:**
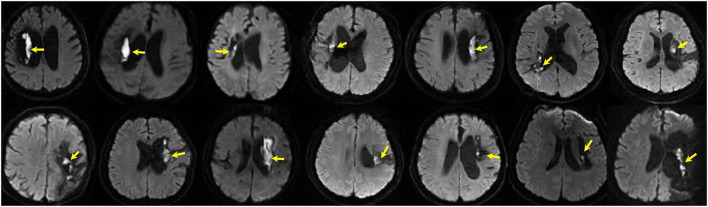
DWI hyperintensities (arrows) at previous hematoma in 14 patients of intracranial hemorrhagic with surgery (ICH–WS) at the chronic stage.

We retrieved 46 patients with ICH–WS who underwent an MRI examination in our hospital between January 2015 and February 2022. After excluding 15 cases who only underwent MRI examination within 3 months after disease onset and 8 cases who had secondary causes as described in the methods section, 23 patients with ICH–WS were finally included in this study. All the patients with ICH–WS underwent endoscopic-guided hematoma removal. Over half (14/23, 60.9%) of the included patients with ICH–WS presented as DWI hyperintensities, with low ADC intensity, and high T1 and T2 intensity at the site of the previous hematoma cavity. The DWI lesions were almost adjacent to the cavity wall of the previous hematoma close to the lateral ventricle. Most of the adjacent cavity walls were rather thin. The DWI lesions were long and narrow or dot-like in shape (Figure 1). In total, two patients with DWI (+) and one patient with DWI (–) had undergone decompressive craniectomy. The age, sex constitution, risk factors, prevalence of epilepsy, hematoma volume, bleeding amount during surgery course, surgery hospitals, interval from disease onset to surgery, and MRI scans yielded no significant differences between patients with DWI (+) and DWI (–). Patients with DWI (+) were more associated with hematoma in the basal ganglia area than in the brain lobes (*P* = 0.02). Significantly more patients with DWI (+) were accompanied by intraventricular hemorrhage during the acute stage than patients with DWI (–) (*P* = 0.02) (Table 1).

**Table 1:** Clinical and imaging characteristics of DWI (+) and DWI (–) patients of ICH-WS.

	**DWI(+)**	**DWI(–)**	**OR(95%CI)**	***p***
Age	58.6 ± 13.9	47.3 ± 17.9	NA	0.08
Sex (M/FM)	13/1	8/1	1.63 (0.09–29.41)	1.00
**Risk factors**				
Hypertension	13 (92.9%)	7 (77.8%)	3.71 (0.28–48.55)	0.54
Diabetes	5 (35.7%)	3 (33.3%)	1.11 (0.19–1.49)	1.00
hyperlipidemia	0	1 (11.1%)	NA	0.39
Smoking	7 (50.0%)	3 (44.4%)	1.25 (0.23–6.72)	1.00
Renal dysfunction	1 (7.1%)	1 (11.1%)	0.62 (0.03–11.28)	1.00
Epilepsy	3 (21.4%)	3 (33.3%)	0.55 (0.08–3.59)	0.64
Hematoma location			12.1 (1.56–143.43)	0.02
Basal ganglia	12 (85.7%)	3 (33.3%)		
Lobar	2 (14.3%)	6 (66.7%)		
Intraventricular hemorrhage	8 (57.1%)	0	NA	0.02
Hematoma volume (ml)	42.5 (39.3–75.0)	42.0 (30.0–62.5)	NA	0.68
Interval from ICH onset to surgery (hours)	8.0 (6.0–12.0)	7.0 (4.5–11.0)	NA	1.00
Interval from ICH onset to MRI scan (months)	6.5 (3–10.75)	10 (6–25.5)	NA	0.23
Bleeding volume during surgery course (ml)	75 (27.5–200)	100 (32.5–600)	NA	1.00
Surgery place			1.07 (0.20–5.77)	0.94
In our hospital	8 (57.1%)	5 (55.6%)		
In other hospitals	6 (42.9%)	4 (44.4%)		

ICH-WS, intracerebral hemorrhage with surgery; M/FM, male/female; DWI (+), diffusion-weighted imaging positive; DWI (–), diffusion-weighted imaging negative; OR, Odds Ratio; 95%CI, 95% confidence interval.

### Multimodal MRI scans of a patient with ICH–WS with DWI hyperintensity

**Figure 2:**
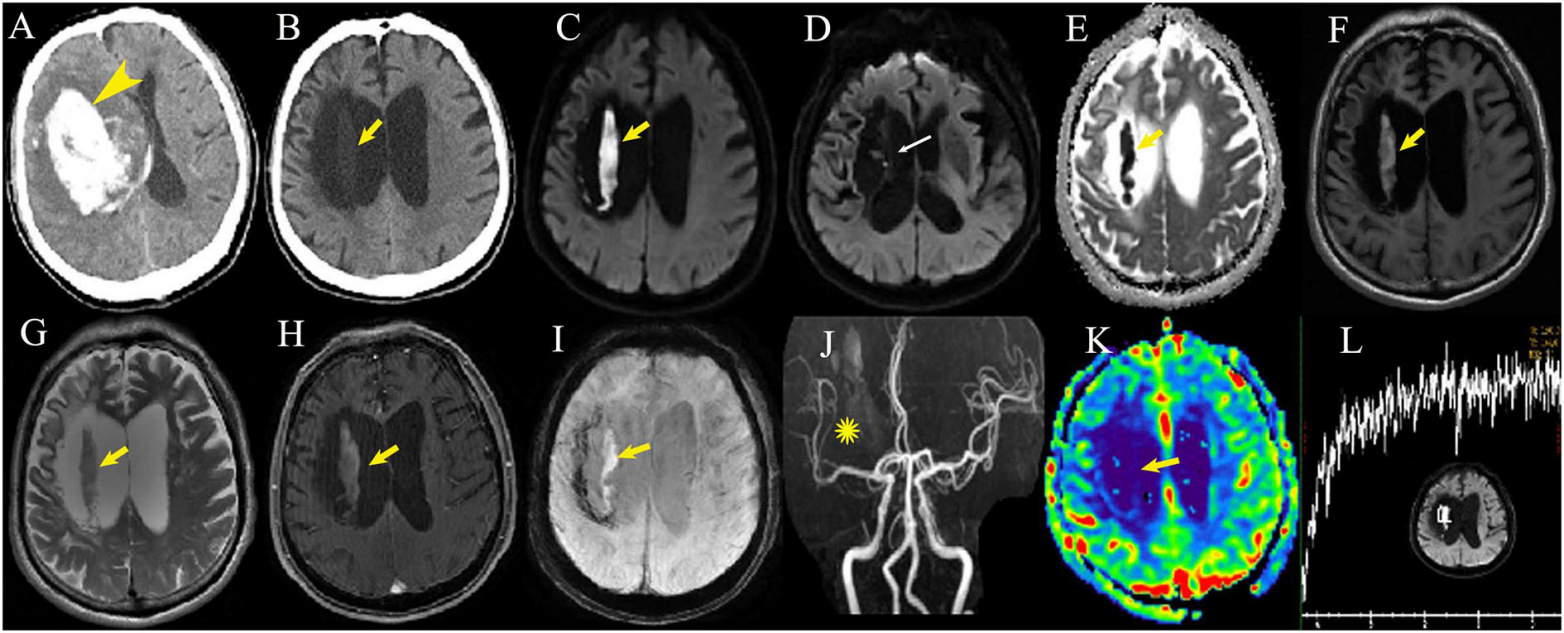
Multimodal imaging examination of one patient 23 months after intracerebral hemorrhage with surgery **(B–L)**. A large hematoma can be seen at the basal ganglia in the first CT scan at disease onset (**A**, arrowhead). Post-surgery CT scan displays low density (**B**, arrow) in the corresponding MRI DWI hyperintense lesion (**C**, arrows) within the hematoma cavity adjacent to the cavity wall. The cavity wall close to the lateral ventricle is very thin (**D**, thin arrow). The apparent diffusion coefficient sequence shows the dark area in accordance with the DWI hyperintensity (**E**, arrow). T1 fat-suppressed image (**F**, arrow), T2-weighted image (**G**, arrow) and susceptibility-weighted imaging (**I**, arrow) show mild hyperintensity with no contrast enhancement (**H**, arrow). MR angiography displays sparse artery branches of the right middle cerebral artery (**J**, asterisk) with low-relative cerebral blood volume by perfusion-weighted imaging (**K**, arrow). No identifiable spectrum of cerebral parenchyma can be seen by magnetic resonance spectroscopy at the DWI lesion **(L)**.

**Figure 3:**
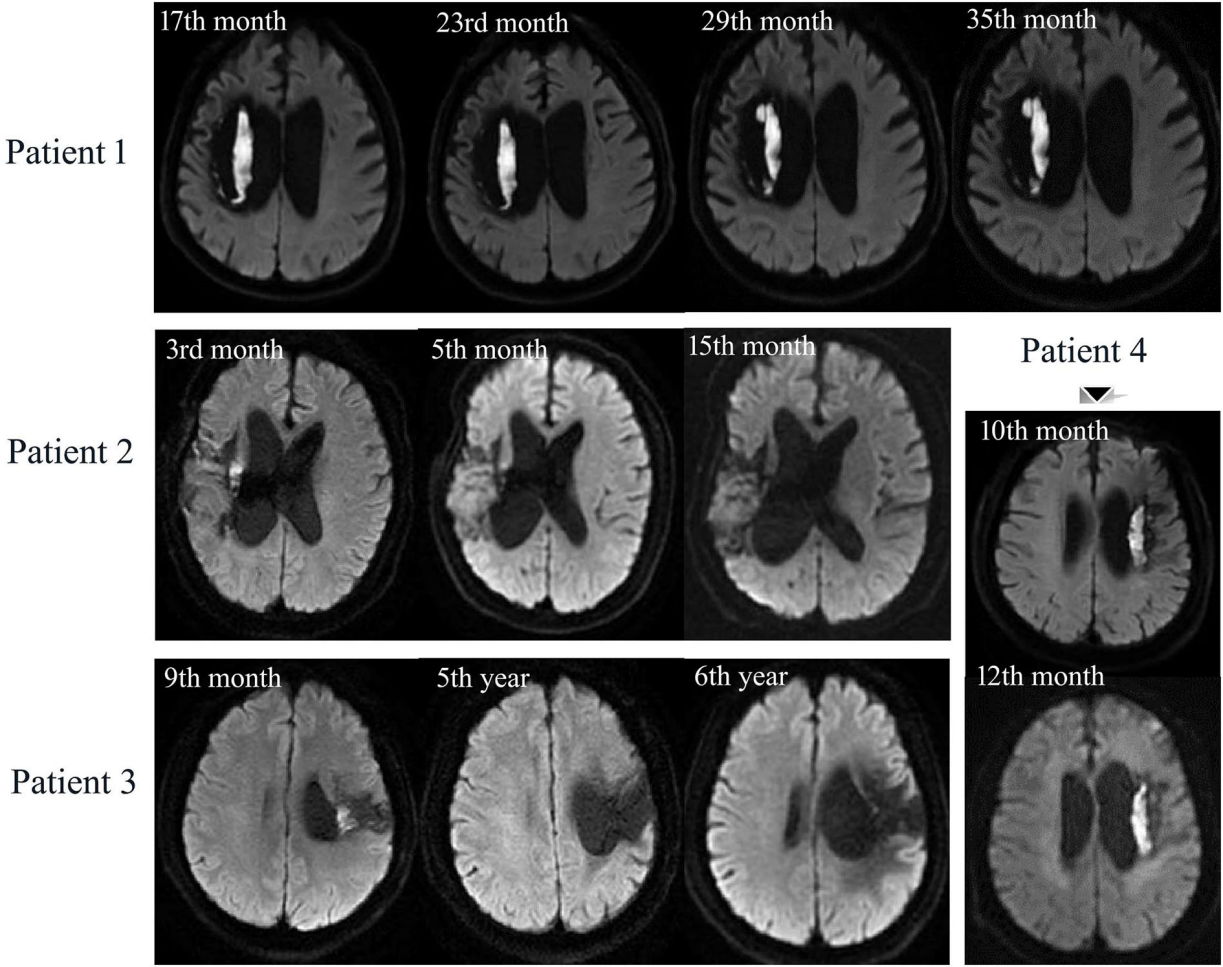
Longitudinal MRI scans of 4 DWI (+) patients with ICH–WS. Patient 1 has persistent DWI lesion during 18 months follow-up. Patient 2: DWI lesion is observable at the 3rd month, shrinks at the 5th month, and disappears at the 15th month. Patient 3: DWI lesion can be seen at the 9th month but disappears at the 5th and 6th year after ICH–WS. Patient 4 has an unchanged DWI lesion during 2 months follow-up.

One 72-year-old male patient presented to our emergency department because of new-onset epilepsy twice in the evening 17 months after ICH–WS. Brain MRI showed a hyperintense DWI lesion at the previous ICH location, and he was admitted to our department with a suspected diagnosis of newly occurring late subacute ICH. The patient had no exacerbated neurological symptoms or findings on physical examination. The DWI lesion manifested as low density on simultaneous CT scan and slightly hyperintense on both T1WI and T2WI, which showed no contrast enhancement, no iron deposition on susceptibility-weighted imaging (SWI), low blood perfusion on perfusion-weighted imaging (PWI), and no identifiable spectrum of cerebral parenchyma by magnetic resonance spectroscopy (MRS) (Figure 2). After receiving sodium valproate, the patient had no epilepsy attacks. In total, three follow-up multimodal MRI scans were performed every 6 months but found no changes in the hyperintense DWI signal (Figure 3-Patient 1).

### Longitudinal observation of DWI lesions in ICH–WS

In total, four of the patients with DWI (+) ICH–WS had MRI scans more than once after 3 months of disease onset (Figure 3). For the two prospectively followed-up patients, Patient 1 had persistently unchanged DWI hyperintensity at the 17th, 23rd, 29th, and 35th months after ICH–WS onset while the other one (Patient 4) also had persistent DWI hyperintensity from the 10th to 12th months. Another two patients had smaller DWI lesions as found at the 3rd and 9th months, respectively, but disappeared after one or more years (Patients 2 and 3).

## Discussion

This study found that DWI lesions were common in patients with ICH–WS 3 months after disease onset and may be persistent for years in some patients. The DWI lesions were located within the previous hematoma cavity, which demonstrated peculiar shape and signals intensity at different MRI sequences. The DWI lesions most frequently occurred in patients with ICH–WS with hematoma in the basal ganglia area and in those accompanied by intraventricular hemorrhage.

The DWI hyperintensity is caused by the restricted diffusion of water molecules with ADC hypointensity and is very sensitive to detecting acute cerebral infarction. Moreover, it may be observed in other situations, such as high viscosity of abscess cavity filled with proteinaceous fluid, high cellularity of malignant tumor, vacuolization, and compartmentalization of water affecting water diffusion (9). Lesions with long T2 relaxation times sometimes have artifactual high-DWI signals with no accordingly ADC hypointensity, which is called the T2 shine-through effect (9). The DWI hyperintensities of our patients presented as low signals on ADC, indicating real water-restricted diffusion of water molecules rather than T2 shine-through effects.

The first glance of DWI lesions in these patients with ICH–WS would mislead doctors to consider them newly occurring cerebral infarction or hemorrhage. Some DWI lesions would even be considered as intracranial infection (10) or periictal lesions (11), as patients with previous ICH–WS may undergo an MRI scan due to fever or epilepsy. However, T1 hyperintensity, as shown in this study, is not the manifestation of acute ischemic stroke, abscess fluid, or periictal lesions but would be present at subacute ICH (12), protein-containing lesions, fatty components, calcification or ossification, lesions with other mineral accumulation, melanin-containing lesions, and miscellaneous lesions (13). Hyperintensity on T1WI of fatty components can be suppressed by T1-fat suppressed (FS) images (14), thus, the hyperintense T1-FS image, as shown in Figure 2F, did not support the attribution of fatty components to T1WI hyperintensity in our patients. Hyperintensity on T2WI in our patients would exclude calcification, mineral accumulation, and melanin-containing lesions as they were usually hypointense on T2WI (15–17). Miscellaneous lesions with T1WI hyperintensity were also seen in ectopic neurohypophysis, chronic phase of multiple sclerosis, and neurofibromatosis type I (13), while these diseases were hard to consider in our patients regarding the peculiar location in close relationship with the previous hematoma.

After excluding other possible pathophysiological mechanisms in forming DWI hyperintense lesions with hyperintensity on T1WI and T2WI sequences, hemorrhagic lesions at the late subacute stage would be therefore considered (2, 12, 18). At the late subacute stage of hematoma, paramagnetic methemoglobin is released by lysis of red blood cells, which cancels the magnetic gradient between the intracellular and extracellular compartments, therefore presenting as short T1 and long T2 signals. In addition, the intracellular contents are distributed in the extracellular space, possibly causing high viscosity and therefore presenting as DWI hyperintensity (2, 12). CT density at the late subacute stage gradually decreases following the lysis of red blood cells and manifests as low density if the hematoma volume is not large (12, 19). However, there were some differences between the features of an intracranial hematoma at the late subacute stage and the DWI lesions of patients with ICH–WS in this study. MRI intensity and CT density of hematoma should not always be homogeneous and are usually accompanied by peripheral edema because of the different progress of hematoma breakdown from the periphery to the center (20), whereas the DWI lesions in our patients were relatively homogeneous without any sign of a core or rim. Furthermore, the shape of a nonchronic hematoma mass would often be oval or round due to hemorrhagic pressure on peripheral brain tissue, but most DWI lesions in our patients were long and narrow in shape or clustered dot-like. Hemorrhagic transformation of cerebral infarction might also be considered at first glance for these DWI lesions. However, most hemorrhagic transformations are smaller than the field of the ischemic infarct (12), whereas the DWI lesions in ICH–WS were relatively homogeneous. Last but most important, the longitudinal follow-up findings of unchanged DWI signal for two patients would further exclude the possibility of recently occurring hemorrhagic lesions.

Multimodal MRI sequences provided more information for DWI lesions. MRS demonstrated no identifiable spectroscopy at the DWI lesion in our patient, indicating that there was no cerebral parenchyma at the DWI lesion (8). PWI with low blood volume indicated that there was little or no blood supply to the lesion (21). Therefore, we supposed that the DWI lesions in patients with ICH–WS might be postsurgical artifacts after removing the hematoma.

Minimally invasive surgery (MIS) for ICH in several randomized controlled studies has been confirmed to be superior to conventional craniotomy (22). Endoscopic surgery, as an MIS, has been widely performed in recent years (23). During endoscopic surgery for intracerebral hemorrhage, the hematoma was gradually evacuated by mild suction under a neuroendoscope, and the hematoma cavity was irrigated with saline. Electrocautery is often used for active bleeding, and absorbable hemostatic gelatin or gauze is usually placed on the wall of the hematoma cavity, which is absorbed within several months (24, 25). We speculated a hypothesis that hemostatic materials absorb permeated blood to form an “island,” and the blood breaks down to extracellular methemoglobin without adequate macrophages from peripheral brain tissue to convert it into hemosiderin and ferritin (12, 26); thus, an extracellular methemoglobin “island” emerges as a prolonged DWI lesion. As shown in this study, the DWI lesions were usually located in the cavity adjacent to the thin wall after basal ganglia ICH and in those accompanied by intraventricular hemorrhage, which indicated that permeating bleeding would more often occur in the deep brain tissue and may suggest that the adjacent wall was too thin to provide enough macrophages to process the extracellular methemoglobin. Intraventricular hemorrhage tends to be more common in deep than lobar hemorrhage with a thinner and broken hematoma wall close to the ventricle (27). For the two followed-up patients with unchanged DWI lesions, the cavity wall of the previous hematoma close to the lateral ventricle was rather thin (Figure 2D), whereas the two patients with disappeared DWI lesions after years presented smaller DWI lesions adjacent to a thicker cavity wall with more residual cerebral parenchyma. Therefore, smaller DWI lesions with thicker adjacent residual cerebral parenchyma would be thoroughly absorbed over a long time. Whether larger DWI lesions with the very thin adjacent walls would be absorbed at last is unknown yet. This hypothesis of an extracellular methemoglobin “island” may be identified at autopsy or by regular MRI follow-up after surgery given that postoperative permeating bleeding occurred after the hemostatic material was placed. More advanced imaging methods such as quantitative susceptibility imaging (28) and positron emission tomography scans may help verify our hypothesis and make a differential diagnosis.

This current study has several limitations. First, the retrospective data from the MRI database would produce selection bias. Second, the results might be influenced by inadequate statistical power due to the small sample size as MRI is not a routine workup for patients with ICH. Only four patients had repeated MRI scans, of whom only two had prospective MRI scans. Due to the limited data of repeated MRI scans, the thorough evolution of the DWI lesion in patients with ICH–WS would not be demonstrated. Third, we did not include patients at the chronic stage who only underwent MRI scans between 1 and 3 months of onset, which also produced selection bias. We chose patients at chronic stage >3 months rather than >1 month in this study because hematoma might demonstrate DWI hyperintensity at the early chronic stage with mixed MRI presentation of late subacute and chronic stage (2). Therefore, future prospective larger-sample studies with regular MRI scans from acute to chronic stages are needed to verify the prevalence and evolution of DWI lesions in ICH–WS.

## Conclusion

Our data revealed a high prevalence of DWI lesions within the previous hematoma cavity during the chronic stage of ICH–WS. The hyperintense DWI signal, with T1WI and T2WI hyperintensity, may last for years or more and also may disappear after years based on the size of the DWI lesion and the thickness of the adjacent hematoma wall. We hypothesized that this extracellular methemoglobin “island” is the possible mechanism of long-lasting DWI lesions in patients ICH–WS as a post-surgery product. Unnecessary further examinations or treatment would be avoided by realizing this imaging phenomenon.

## Data availability statement

The original contributions presented in the study are included in the article/supplementary material, further inquiries can be directed to the corresponding authors.

## Ethics statement

The studies involving human participants were reviewed and approved by the Local Ethics Committee of Chinese PLA General Hospital. The patients/participants provided their written informed consent to participate in this study. Written informed consent was obtained from the individual(s) for the publication of any potentially identifiable images or data included in this article.

## Author contributions

XC designed the study, developed the protocol, collected and analyzed data, and wrote the manuscript. SG and YL developed the protocol, collected and analyzed data, searched for literatures, and wrote part of the manuscript. XH helped collect data and provided helpful input on the theme. RL, CT, and RC helped build the idea, analyze, and interpret the data. ZD assisted with the study design, developed the protocol, analyzed data, and revised the manuscript. SY assisted with the study design, analyzed data, and offered available suggestions to write the manuscript. All authors contributed to the article and approved the submitted version.

## Conflict of interest

The authors declare that the research was conducted in the absence of any commercial or financial relationships that could be construed as a potential conflict of interest.

## Publisher's note

All claims expressed in this article are solely those of the authors and do not necessarily represent those of their affiliated organizations, or those of the publisher, the editors and the reviewers. Any product that may be evaluated in this article, or claim that may be made by its manufacturer, is not guaranteed or endorsed by the publisher.
